# The Lesson Learned from the COVID-19 Pandemic: Can an Active Chemical Be Effective, Safe, Harmless-for-Humans and Low-Cost at a Time? Evidence on Aerosolized Hypochlorous Acid

**DOI:** 10.3390/ijerph192013163

**Published:** 2022-10-13

**Authors:** Mascia Benedusi, Elena Tamburini, Mariaconcetta Sicurella, Daniela Summa, Francesca Ferrara, Peggy Marconi, Franco Cervellati, Stefania Costa, Giuseppe Valacchi

**Affiliations:** 1 Department of Neurosciences and Rehabilitation, University of Ferrara, 44121 Ferrara, Italy; 2 Department of Environmental and Prevention Sciences, University of Ferrara, 44121 Ferrara, Italy; 3 Department of Chemical, Pharmaceutical and Agricultural Sciences, University of Ferrara, 44121 Ferrara, Italy

**Keywords:** bactericidal, COVID-19 pandemic, hypochlorous acid, indoor sanitization, virucidal

## Abstract

The COVID-19 pandemic has underlined the importance of disinfectants as tools to prevent and fight against coronavirus spreading. An ideal disinfectant and sanitizer must be nontoxic to surface contact, noncorrosive, effective, and relatively inexpensive as it is hypochlorous acid (HOCl). The present work intended to evaluate, on different surfaces, the bactericidal and virucidal effectiveness of nebulized HOCl and test its safety usage in 2D and 3D skin and lung models. Our data showed that HOCl at the dose of 300 ppm did not affect cellular and tissue viability, not their morphology. The HOCl bactericidal properties varies with the surface analyzed: 69% for semi-porous, 96–99.9% for flat and porous. This discrepancy was not noticed for the virucidal properties. Overall, this study showed that nebulized HOCl can prevent virus and bacteria growth without affecting lung and skin tissues, making this compound a perfect candidate to sanitize indoor environments.

## 1. Introduction

The COVID-19 pandemic has had a significant impact on both physical health and psychological well-being of communities and is gripping the world through its devastating pandemic [1].

Conversely, it has also contributed to reinforce the awareness of the importance of defending us against the spread of infectious diseases and effectively tackling their occurrence [2]. While it has been dominating the media since 2020, COVID-19 is not the only outbreak that has been causing concern worldwide, especially in low-income countries. In fact, besides COVID-19, newly emerging or re-emerging viruses and microbial infections are now the top causes of death worldwide, especially in young populations [3]. Deaths from infectious diseases are common—and in some cases dominant—across low- and middle-income nations. On the other hand, healthcare-associated infections have been becoming a major problem in industrialized countries, with 1.7 million cases, and an estimated 100,000 deaths, per annum reported in the US alone [4].

COVID-19 has undoubtedly placed hygiene at the center of disease prevention and has challenged basic hygiene behavior as hand washing and surfaces disinfection in the daily routine. As described by Enyoh and colleagues [5] the virus persistence time on different inanimate surfaces differs from 24 h (aluminum or surgical gloves) to more than 5 days (ceramic, Teflon, silicon, or paper) depending on the type of surface, but in any case, it represents one of the main issues in the spreading of COVID-19 infection. For this reason, during the COVID-19 pandemic, in all the high-risk public spaces, such as schools, healthcare facilities and workspaces, the global consumption of sanitizers and disinfectants has unprecedently been raising [6], to the extent that the global disinfection market size has been estimated to be worth USD 105 billion by 2028, growing at a CAGR of 20.8% from now on [7].

Otherwise, a substantial concern about the possible adverse effects of disinfectants has also been raised [8]. Indeed, when abused or misused, disinfectants can also be hazardous to humans as well as to the environment and may drive to worldwide secondary global noxious consequences for human health and ecosystems [9,10]. Common disinfectants, such as sodium hypochlorite, ozone, oxygen peroxide, and nanomaterials, are also known to cause irritation of the skin, eyes and to the respiratory tract [11,12].

Currently, an eco-friendly disinfectant acting as effective biocide and virucide at a broad spectrum of activity, while causing minimal or no harm to the environment, prepared in a way to avoid waste, with a very low toxicity, safe and easy to handle and store, would be strongly needed [13]. Moreover, disinfectants and disinfection techniques used in the fight against infectious agents should be safe for a long exposure time on personnel, equipment, and surfaces. Disinfection technologies for indoors are important to reduce transmission, especially in crowded settings [14].

Hypochlorous acid (HOCl) could be proposed as a promising candidate to fulfill that role. There is wide literature demonstrating its efficacy against a broad range of microorganisms, including spores and viruses [15]. HOCl is an endogenous substance in all mammals, usually produced by white blood cells to surround pathogens, e.g., when the skin is injured, and pathogens begin to invade [16]. Its mechanism of action relates to the destruction of the cytoplasmic membrane and the cell wall of the bacteria, due to its small molecular dimensions, such as the water molecule, and neutral charge, not repelled by bacteria [17]. For decades, chlorination has been securing the microbiological quality of drinking water [18]. One of the recent advancements in chlorine-based disinfection is its utilization in the form of electrolyzed water (EW). This method became attractive due to its proven simplicity, biocidal efficiency, and convenience of in situ production, which reduces the hazard of handling and transporting concentrated chlorine reagents [19]. EW is usually produced by passing a dilute salt solution (NaCl) through an electrolytic cell, subjected to a DC voltage. The spontaneous reduction of hypochlorous acid to chloride, after disinfection process via oxidation, accelerated if exposed to light and air, assures the absence of residual effects or damages on environment [20].

In recent years, while EW liquid solution has been widely accepted for disinfection by different sectors, such as medical, agricultural, food processing and sanitary industries [21], its application as aerosolized dry fog is still barely known and few studies have investigated its potentiality for indoor environments and surfaces disinfection [22]. The exposure of individuals to microbes can take place via inhalation or through contact with contaminated surfaces and objects and is allowed by the transport of the species, in the form of bioaerosol, either by binding to dust or particles present in the air, or through individuals themselves. In particular, the contamination of surfaces, which act as reservoirs for microorganisms, is a crucial point in the indirect transmission of infections [23]. Starting from this evidence, here we describe the experimental proof of antimicrobial and antiviral activity of neutral HOCl solution aerosolized on different surfaces (flat, porous, and semi-porous) and at different times of indoor treatments. We have started this study on the development of a smart prefabricated disinfection chamber (SPSC) by way of aerosolized hypochlorous acid at 300–10,000 ppm concentration interval. Then, by means of a 2D model of human keratinocytes cell line (HaCaT cells), we assessed the optimal dose of aerosolized HOCl to be used in the next experiments.

The choice to use different surfaces for our study is therefore due to the different ability of microorganisms (bacteria and viruses) to adhere to the surface, but also to the different nature of the materials. At this point we considered it important to evaluate the capacity and effectiveness of HOCl in nebulized form on different surfaces (semi-porose, flat and porose), contaminated by bacteria and viruses. To be considered a good disinfectant, the product must have a broad-spectrum action [24,25].

HOCl was tested in *E. Coli*, *S. Aureus* and *P. Aeruginosa* as a bacterial model, as *E. coli* and *P. aeruginosa* bacteria can survive in conditions of high humidity, unlike the *S. aureus* bacterium which prefers drier environments, as well as being able to survive even at low temperatures, from 4 °C to 6 °C [26]. Viruses also can resist various environmental factors and disinfection techniques, even those that are prolonged over time. The absence of an envelope (Adenovirus V) gives them greater resistance to drying, making them more stable in the environment. While enveloped viruses, such as respiratory viruses (coronavirus), usually remain infectious on surfaces for hours or days [27]. Finally, the toxicity of the selected dose of HOCl on human cell-based 3D tissues was evaluated; in particular, we selected two of the principal target tissues of nebulized drugs: the cutaneous and respiratory tissues.

This study proposes the use of an in situ HOCl-based disinfectant production unit. The ecological consequences of this new system are limited and HOCl does not leave residue on the environmental surface nor in the indoor air and is not corrosive to the equipment and furniture. Even from this limited demonstration study, we proved its efficacy and safety as biocide even in aerosolized form. Important general advantages on its potential application in fighting against the COVID-19 pandemic and other infectious diseases can be extracted by performing a relative comparison with other different disinfection methods.

## 2. Materials and Methods

### 2.1. Preparation of HOCl Solution

HOCl solution was prepared by electrolysis of a mixture of aqueous dilute solutions of NaCl at 30,000 ppm and deionized water added with a salt-based buffer (Na_2_SO_4_, 7000 ppm) for maintaining pH at slightly acid value (about 6.5). The EW generator works at 12.0 V and 50.0 A for 3 h, consuming 0.06 kWh/L. It basically consists of an electrolytic cell with anode and cathode electrodes, without a separating membrane between them. The two electrodes are composed of a titanium core coated with iridium or platinum oxide, without polarity inversion. During electrolysis Na^+^ ions will be directed to the cathode, while Cl^−^ ions will approach the anode, where hypochlorous acid (HOCl) is formed and released in solution as the prevalent species (>95%), when pH is maintained within the 6.0–6.7 interval. HOCl concentration was determined using the DPD colorimetric test method.

HOCl solution has been produced at 10,000 ± 250 ppm of HOCl and subsequently diluted with deionized water at 1:2 (5000 ppm), 1:5 (2000 ppm), 1:10 (1000 ppm), 1:33 (300 ppm) and 1:100 (100 ppm). HOCl solutions have been stored in the dark at 4 °C.

### 2.2. HOCl Solutions Aerosolization

HOCl solutions have been transferred into a nebulizer, equipped with a tank (24 L of working volume) connected with two stainless steel nozzles by means of a HDPE pipe (length, 3 m). Nozzles have been designed to ensure a constant release in the environment of a dry fog of 1 mm of droplet diameter. Hydraulic pump pressure was 0.4 bar and the volumetric flowrate 100 ± 20 mL/min. The aerosolization has been performed using a specific plexiglass system specifically designed for this experiment (patent 102022000002699 pending). Chamber saturation was reached in 3 min, aerosolizing 300 mL of HOCl solutions at different concentrations.

### 2.3. 2D Human Keratinocytes and Cell Viability

The spontaneously immortalized human keratinocyte HaCaT cell line was used for the cellular experiments. HaCaT cells were cultured as previously described [28] and exposed to different doses of HOCl (from 50 ppm to 10,000 ppm) for 30 min. At the end of the exposure, the cells were harvested and analyzed at different time points; in particular, cell viability was evaluated by Trypan blue staining. After the exposure to HOCl cells were trypsinized and resuspended in 1 mL of culture media. Next, 10 µL of Trypan blue solution (Sigma, Milan, Italy) was added onto 10 µL of cell suspension and viable cells were counted in a Bürker cell counting chamber at each time point. After the addiction of Trypan blue solution, the non-viable cells appeared blue. Viable and non-viable cells were counted separately, and the cell viability was expressed as percentage of living cells with respect to the total number of cells:

viable cells (%) = (total number of viable cells per mL/total number of cells per mL) × 100 as previously demonstrated [29].

### 2.4. MTT Assay on 2D Human Keratinocytes

HaCaT cells were plated (2.0 × 10^4^ cells/well) in 96 well multiplates in DMEM with 10% FBS. After 24 h, the medium was removed, and cells were incubated for 4 h with fresh medium in the presence of 1.2 mM MTT (3-(4,5-dimethylthiazol-2-yl)-2,5-diphenyltetrazolium bromide) (Sigma-Aldrich, St. Louis, MO, USA). After that, the MTT solution was removed and 100 µL of DMSO was added to each well to dissolve the blue formazan crystals. The absorbance of the formazan dye was measured at 570 nm with a microplate reader (Spectramax M5 multimode microplate reader, Molecolar Device, San Jose, CA, USA). Data were expressed as a percentage of the control value as previously demonstrated [30].

### 2.5. Scanning Electron Microscopy (SEM) Methods on 2D Human Keratinocytes

Sample morphology, size and distributions were evaluated by observation by optical and electron microscopy. To analyze the external morphology, the cells were fixed in 2.5% glutaraldehyde in cacodylate buffer for 3 h at 4 °C. Then, cells were post-fixed in 1% osmium tetroxide for 2 h at 4 °C and dried in a graded series of alcohol; samples were then metalized by gold coating (Edwards Sputter Coating S150) and analyzed at 15–20 Kv by a scanning electron microscope (Zeiss EVO 40) source LaB6.

### 2.6. Transmission Electron Microscopy (TEM) Methods on 2D Human Keratinocytes

For ultrastructural analysis, treated HaCaT cells were fixed in 2.5% glutaraldehyde in cacodylate buffer for 3 h at 4 °C. Then, cells were post-fixed in 1% osmium tetroxide for 2 h at 4 °C, dehydrated in a graded series of alcohol, embedded in Araldite resins, and polymerized in oven for 48 h at 60 °C. Ultrathin sections of 60 nm were cut with an ultramicrotome (Ultratome Reichert SuperNova Leica, Wien, Austria), stained with uranyl acetate and lead citrate and examined in a Philips CM100 transmission electron microscope.

### 2.7. 3D Skin Model and Epiairway Model

A 3D skin model “EpiDerm” (reconstructed human epidermis (RHE)) and 3D Epiairway model were purchased from MatTek corporation (MatTek In Vitro Life Science Laboratories, Bratislava, Slovak Republic). Upon arrival, the 24 inserts containing 3D tissues were transferred into 6-well plates prefilled with 1 mL of specific MatTek Assay medium (provided by MatTek Corporation, Ashland, MA, USA), according to the manufacturer’s instructions.

The tissues were kept at 37 °C, 5% CO_2_ atmosphere overnight. On the day of the experiment, fresh media were added, and the tissues were exposed to 300 ppm HOCL for 30′ in the aforementioned box. For the Epiairway tissues, the apical surface was rinsed with PBS to eliminate accumulated mucus, then fresh medium was added immediately before the experiment [31]. Control tissues were left in the incubator. After exposure, tissues were immediately processed (time point T0) or after 24 h (time point T24).

### 2.8. Cytotoxicity Determination (LDH Assay)

Cytotoxicity was assessed by lactate dehydrogenase (LDH) leakage in the culture medium, consequent to loss of the plasma membrane integrity. LDH activity was determined by measuring the conversion of pyruvate to lactate via NADH oxidation (the rate of NADH consumption is proportional to LDH activity). Cell-free culture medium (40 µL) was collected for each sample and added to 160 μL of the assay mixture (substrate buffer:NADH buffer, 9:1). The substrate buffer was 1.2 mM sodium pyruvate, 5 mM ethylenediaminetetraacetic acid (EDTA), 13.8 mM sodium azide, in 50 mM Tris buffer, pH 7.4; the NADH buffer was 1.8 mM NADH, 13.8 mM sodium azide, in 50 mM Tris buffer, pH 10.2. LDH activity was measured at room temperature in a microplate reader (Victor3 Multilabel Counter; Perkin-Elmer, Waltham, MA, USA); the absorbance decrease (measuring NADH oxidation) was recorded at 340 nm over a period of 8–10 min. In order to obtain a representative maximal LDH release as the positive control with 100% toxicity, a triplicate set of samples were lysed with 2% (*v/v*) Triton X-100 in culture media for 30 min at 37 °C.

### 2.9. MTT Assay on 3D Tissues

Tissue viability was measured by reduction of the tetrazolium salt Methyl Thiazoyl Tetrazolium (MTT). Briefly, a 1 mg/mL of MTT stock solution was prepared in maintenance media (provided with tissues) just prior to use and warmed to 37 °C.

Tissue samples were transferred to 24-well plates containing 300 µL MTT reagent per well and placed in the 37 °C, 5% CO_2_ incubator for 3 h. After that, the tissues were submerged in 2 mL of MTT extract and maintained overnight at room temperature protected from light. The day after, 200 µL of extraction solution was transferred to a clear 96-well plate and the optical density was measured with a spectrophotometer at 570 nm. Finally, the cell viability was calculated as percent of the mean of the control tissue.

### 2.10. Measurement of TEER

Tissues barrier integrity was evaluated by TEER measurements using a Millicell-ERS 2 V-Ohmmeter (Millipore Co., Bedford, MA, USA) at T0 and T24 after 300 ppm HOCL nebulization. For each well, 3 measures were performed. Briefly, the tissues were equilibrated for about 20 min at room temperature before the measurements. Blank measurements were performed on an empty insert. TEER value was then calculated using the following equation: TEER (Ωcm^2^) = (Rsample—Rblank) × membrane area (cm^2^).

### 2.11. Histological Analysis

For this step, 3D tissues, exposed or un-exposed to 300 ppm HOCL, were immersion-fixed in 10% NBF (neutral-buffered formalin) for 24 h at room temperature, then dehydrated in alcohol gradients and embedded in paraffin. For histological observation, the sections (4 µm thickness) were deparaffinized in xylene and rehydrated in alcohol gradients (100%, 90%, 80% 70%). The sections were stained with hematoxylin for 3–5 min, washed in running tap water for 5 min, and stained with Eosin Y for 2 min. The sections were then washed in tap water for 1–5 min, dehydrated in increasing concentrations of alcohols and cleared in xylene. The sections were then mounted with a rapid non aqueous mounting medium, containing toluene (Entellan, Merck KGaA, Darmstadt, Germany) and observed under Nikon Microphot FXA microscope (Nikon Instruments, Amsterdam, The Netherlands).

### 2.12. Bacterial Culture

Bacteria used for this study were purchased from the ATCC company, *Escherichia Coli (ATCC 8739)*; *Pseudomonas Aeruginosa (ATCC 9027)*; *Stafilococco Aureus (ATCC 6538)*. Supplied in dry pellet and subsequently resuspended in tryptic soy broth. Bacterial cultures and experimental procedures were executed in a biosafety level 2 laboratory, designed and managed in accordance with safety regulations. Liquid cultures were grown for 18 h in Tryptic soy broth at 37 °C.

### 2.13. Cell Culture Used for Virucidal Assay

The human lung fibroblast cell line MRC-5 was supply by (LGC Standard-ATCC CCL171). MRC-5 cells were maintained in Eagle’s Minimum Essential Medium (EMEM) ATCC 30-2003, supplemented with 15% FBS (Euroclone), 1% penicillin-streptomycin solution 100X (Euroclone) and 1% non-essential-amino acid (Euroclone).

For the human cancer cell line HeLa (LGC Standard-ATCC CCL-2), cells were cultured in RPMI-1640 medium (Euroclone), with 10% FBS, 1% penicillin–streptomycin solution 100X (Euroclone). Cells were incubated at 37 °C in 5% CO_2_.

### 2.14. Growth of Virus

Human coronavirus strain 229E and Human Adenovirus V were supplied by (LGC Standard-ATCC Coronavirus 229E VR-740; Adenovirus V VR-5). MRC-5 or Hela monolayers were infected with 229E or Adenovirus V at a multiplicity of 0.1 MOI and were incubated in medium with 2% serum, supplemented with glutamine, antibiotics and nonessential amino acid at 33 °C—5% CO_2_ incubator for until complete cytopathic effect (CPE) is reached. Cells and supernatant were collected, cells removed by low-speed centrifugation and the supernatant was centrifugated a 20,000 rpm for 30 min. The pellet obtained was resuspended in small aliquots and kept at −80 °C until use.

### 2.15. Bactericidal Activity by Nebulization

This test involves the nebulization of the EW solution (300 ppm) on three different surfaces (semi-porous-flat and porous) previously contaminated with the bacterial suspension. On the surfaces, areas to be contaminated were indicated, all of the same size, in order to minimize variability. After contamination, the surfaces were placed inside the plexiglass chamber and subjected 10 min of exposition of nebulization The test includes a negative control represented by the sample contaminated with the bacterial culture and not subjected to nebulization. After treatment with EW solution, swabs were carried out on the surfaces to evaluate the residual bacterial colonies. These swabs were resuspended in medium and subsequently serial dilutions were made and seeded on agar plates. The plates were incubated at 37 °C for 24 h and checked to assess the presence or absence of bacterial colonies.

The calculation of antibacterial activity was carried out according to the formula:

R = (Ut − U_0_) − (At − U_0_) = Ut − At

where:

R is the antibacterial activity;

U_0_ is the average of the common logarithm of the number of viable bacteria, in cells/cm^2^, recovered from the untreated test specimens immediately after inoculation;

Ut is the average of the common logarithm of the number of viable bacteria, in cells/cm^2^, recovered from the untreated test specimens after 24 h;

At is the average of the common logarithm of the number of viable bacteria, in cells/cm^2^, recovered from the treated test specimens after 24 h.

### 2.16. Virucidal Activity by Nebulization

Three different surfaces (semi-porous, flat and porous) were used to evaluate the virucidal activity of hypochlorous acid in nebulized form. These surfaces were contaminated with Coronavirus 229E (virus a RNA with envelope) or Adenovirus V (virus a DNA without envelope) and then sprayed for 10 min with hypochlorous acid. After treatment, swabs were carried out on the surfaces to check for the possible presence of viruses after nebulization. Serial dilutions were then carried out on plates previously seeded with the MRC-5 or Hela cell lines. As a control, infection was carried out on the three surfaces not subjected to treatment. The plates were transferred to a 37 °C incubator with 5% CO_2_. In the following days, the plates were checked under a microscope to verify the presence or absence of the virus. The number of infectious viral particles is often quantified using the Median Tissue Culture Infectious Dose (TCID50) assay.

The amount of virus is expressed as TCID50/mL:

TCID50 = D − [d (S − 0.5)]

where:

D = −log10 of the last dilution which has 100% virus positivity (for example 3 wells/3 wells);

d = −log10 of the dilution factor;

S = number of wells in which the virus is present, including the last dilution with 100% positivity.

### 2.17. Statistical Analysis

All experiments were repeated three times and statistical values were expressed as the mean ± standard deviation (SD). A Student’s *t*-test or One-way ANOVA were used. Values of *p* < 0.05 were considered statistically significant. For all data analysis, GraphPad Prism 9 software (GraphPad Software Inc., San Diego, CA, USA) was used.

## 3. Results

### 3.1. Effects of HOCl Nebulization on Human Keratinocytes Viability and Cytotoxicity

To evaluate the effect of HOCl on epidermal tissue we selected the human adult keratinocytes (HaCaT) since they are often used as a model for the study of epidermal physiology [32]. The first step was to investigate the appropriate doses of HOCl to be used in our experimental protocol, i.e., to determine the highest concentration that did not cause death or any obvious morphological defects of keratinocytes.

**Figure 1:**
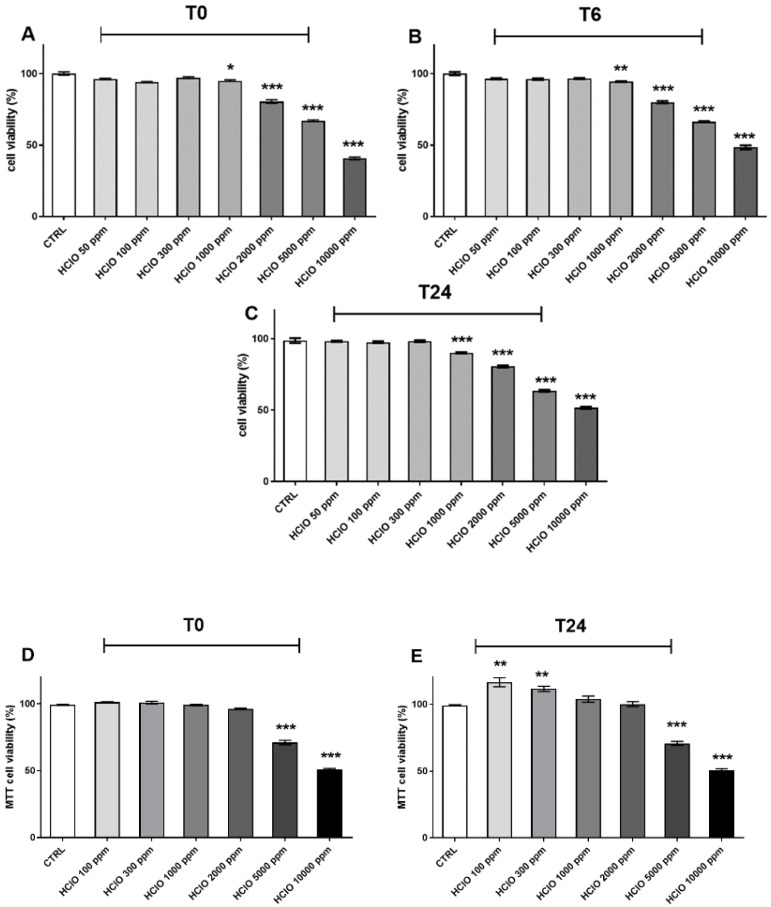
Effects of HOCl nebulization on HaCaT cell proliferation and viability; after the nebulization of different doses of HOCl, cells were collected immediately after the exposure and after 6 or 24 h. Cell viability was assessed by Trypan blue exclusion test (**upper panels**, **A**–**C**) and MTT assay (**below panels**, **D**,**E**). Data are expressed as mean ± SEM of four different experiments. * *p* < 0.05; ** *p* < 0.01; *** *p* < 0.0001 exposed vs. CTRL. Results were analyzed by One-way ANOVA.

As depicted in Figure 1A–C HOCl nebulization did not affect cellular viability from 50 ppm to 300 ppm at all time points. A slight and consistent effect (5–8% decrease) on cellular viability was noticed for 1000 ppm over the time points analyzed (T0 to T24) compared to the control condition. Decrease in cellular viability was even more evident at the higher doses (from 2000 to 10,000), reaching about 60% of mortality for 10,000 ppm at all time points. These results were in line with those obtained with the MTT assay (Figure 1D,E), where we observed a significant decrease in HaCaT metabolic activity, in particular for the higher doses of HOCl (from 5000 to 10,000 ppm) at both T0 and T24 h after the nebulization.

Although very minor, after 24 h (T24) of exposure to HOCl at the lower doses (100 and 300 ppm) there was an increase in the keratinocytes metabolic rate. Overall, these data suggest that up to a dose of 300 ppm, the HOCl nebulization did not affect cellular viability nor cellular metabolic rate.

### 3.2. Effects of HOCl Nebulization on Human Keratinocytes Morphology

**Figure 2:**
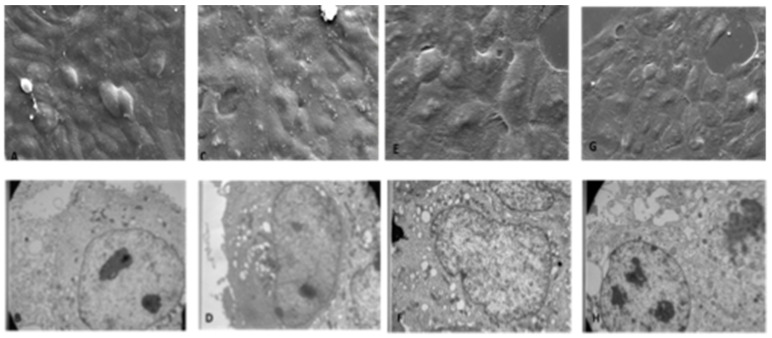
Effects of 100 ppm HOCl nebulization on HaCaT cells morphology analyzed by SEM and TEM microscopies. (**A**) Ctrl T0h; SEM image: morphologically normal cells, in tightly adhered uniform monolayer. (**B**) Ctrl T0h; TEM image: structurally unaltered nuclei and organelles, low percentage of vacuolated cells. (**C**) Ctrl T24h; SEM image: after 24 h the cellular carpet surface remains uniform without variations. (**D**) Ctrl T24h; TEM image: cells have normal cytoplasmic and nuclear structures. (**E**) 100 ppm T0h; SEM image: cells morphologically unchanged compared to controls, in monolayer it remains uniform. (**F**) 100 ppm T0h TEM image: the treatment at this concentration does not alter the cellular structure which remains the same as that observed in the controls. (**G**) 100 ppm T24h; SEM image: after 24 h the cellular carpet surface is still uniform. (**H**) 100ppm T24h; TEM image: cells have cytoplasmic and nuclear structure without relevant alterations. 5K magnification (SEM pictures) and 10K Magnification (TEM pictures).

As depicted in Figure 2A–D, both SEM and TEM analyses did not show morphological alterations in HaCaT cells at both time points (T0 and T24).

**Figure 3:**
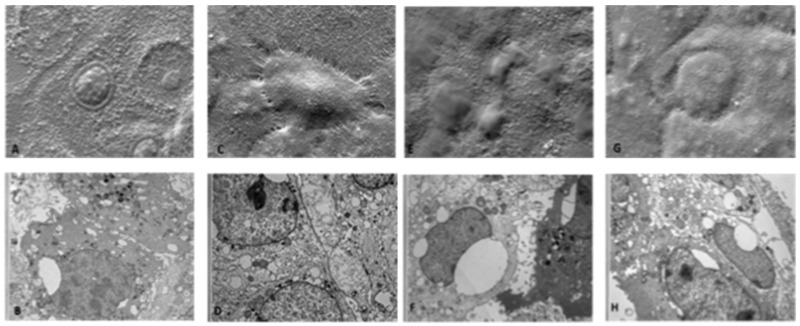
Effects of 300 and 5000 ppm HOCl nebulization on HaCaT cell morphology analyzed by SEM and TEM microscopies. (**A**) SEM image: T0h 300 ppm: SEM images show some cells with pycnotic nuclei and expanded cytoplasm in contact with neighboring cells. (**B**) TEM image T24h 300 ppm: the photomicrograph below at the same magnification shows cells with some vacuolations and lysosomal vesicles. (**C**) SEM image: T24h 300 ppm: SEM images show cells that tend to lose adhesion from contact with neighboring cells, rising from the underlying carpet. (**D**) TEM image T24h 300 ppm: cells with extensive vacuolization, cytoplasmic degranulation and structural loss of organelles are shown. (**E**) SEM image T0h 5000 ppm: the diffuse presence of surface alterations of the membrane appears on the scan. (**F**) TEM image T0h 5000 ppm: the vast vacuolization, the structural alteration of the mitochondria and the presence of a significant number of lysosomal and peroxisomal vesicles. (**G**) SEM image T24h 5000 ppm: the cells appear disrupted and with loss of adhesion. (**H**) TEM image T24h 5000 ppm: confirms the presence of a high percentage of cells in necrosis, pycnotic nuclei and cytoplasmic disaggregation. 5K magnification (for both SEM and TEM pictures).

The cellular ultrastructure was not altered even after treatment with 100 ppm HOCl (Figure 2E–H). Similarly, HOCl 300 ppm treated cells maintained both superficial and intracellular structural characteristics intact (Figure 3A–D).

On the other hand, when the cells were treated with 5000 ppm of HOCl, some superficial alterations were noticed (Figure 3E,G). Intracellular images obtained with the transmission microscope showed evident and relevant structural alterations, with large vacuolizations, loss of intercellular adhesion, presence of dense cytoplasmic deposits at T0 and, a later time point (T24). mitochondrial structures were also altered (Figure 3F,H).

**Figure 4:**
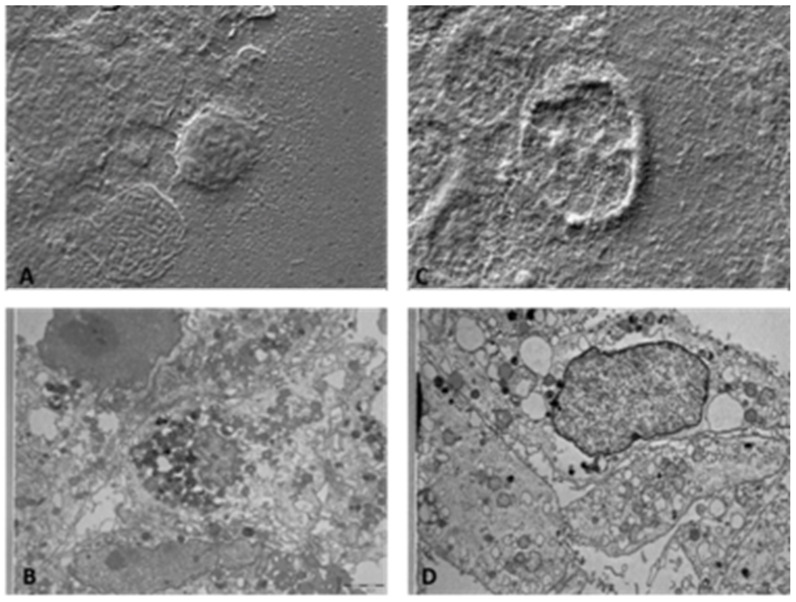
Effects of 10,000 ppm HOCl nebulization on HaCaT cell morphology analyzed by SEM and TEM microscopies. (**A**) SEM imageT0h 10,000 ppm: at maximum conc. of the SEM treatment the cells appear disrupted and with loss of adhesion. (**B**) TEM image T0h 10,000 ppm: confirms the presence of a high percentage of necrotic, pycnotic nuclei and cytoplasmic disaggregation. (**C**) SEM image T24h 10,000 ppm: at this conc. at SEM the cells appear deeply damaged with loss of both cytoplasm and nuclear support. (**D**) TEM image T24h 10,000 ppm: confirms the presence of a widespread percentage of cells in necrosis, pycnotic nuclei and disaggregation of cytoplasm, and there are numerous cells in apoptosis. 5K magnification (for both SEM and TEM pictures).

As depicted in Figure 4, SEM images showed that exposure to 10,000 ppm of HOCl induced an evident cellular damage already at T0 (A) and this effect was even more pronounced at T24 (C). A similar effect was evidenced by TEM (Figure 4B,D) with the high degree of intracellular alteration and the presence of numerous necrotic cells.

### 3.3. Bactericidal Activity by Nebulization

**Figure 5:**
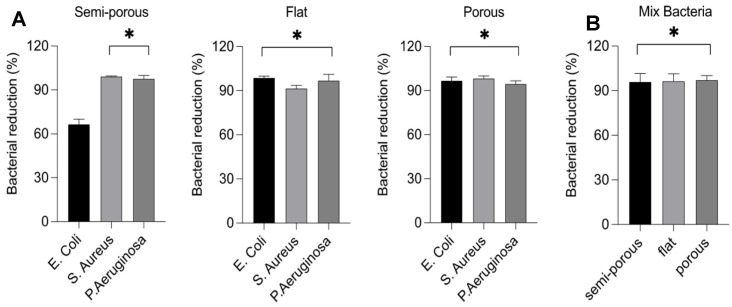
Bactericidal effect of HOCl solution at 300 ppm against *E. coli*, *S. aureus* and *P. aeruginosa* 1 × 10^6^ CFU/mL. The bacteria were incubated in the different surfaces—semi porous, flat and porous. Data represent the percentage of bacterial reduction after 10 min of exposition of nebulization. (**Panel** **A**) shows the single reduction of microbial growth on different surfaces, while (**panel** **B**) shows the reduction growth of a mix of microorganisms. Values were obtained by comparing the results with the bacteria control. Data represent the mean of three independent experiments conducted in triplicate. * *p* < 0.05.

Once the optimal dose of HOCl was selected by the experiment on the 2D cell model, we moved one step further and analyzed how the nebulization treatment with the hypochlorous solution at 300 ppm can effectively reduce microbial growth. The three bacterial models for the study present differentiated structural characteristics that allow us to underestimate the effectiveness of the HOCl solution on both Gram positive and Gram negative. Staphylococcus Aureus was selected for Gram positive, and Escherichia Coli and Pseudomonas Aeruginosa bacteria were selected for Gram negative. Different surfaces were used for the test, semi-porous, flat and porous, contaminated with the individual microorganisms *E. Coli*; *S. Aureus* and *P. Aeruginosa* at 1 × 10^6^ CFU/mL or mix of these microorganisms (Figure 5A). The choice of these materials is linked to their natural diversity, but also to their wide presence in different work environments, while the bacteria were selected for the different ability to survive on various surfaces, resistant even to adverse situations. As shown in Figure 5A it was possible to observe how different materials and the different ability of microorganisms to adhere on inanimate surfaces can influence the effectiveness of the treatment. The range of reduction was 69% for the semi-porous surface, 96–99.9% for flat and porous surfaces, respectively (Figure 5A and Table 1). The same results were obtained by contaminating the surfaces with a mix of 3 × 10^6^ CFU/mL bacteria. In this case, the diversity of materials does not seem to affect the bactericidal capacity of the product, this may be partly due to the ability of microorganisms to interact with each other, as we observed a reduction in bacterial growth of 99.3% for the porous material, while on smooth surfaces, such as semi-porous and flat, the reduction was 99.9% (Figure 5B and Table 2).

### 3.4. Virucidal Activity by Nebulization

The virucidal activity represents the capability of a product to produce a decrease in the number of infection virus particles under defined condition. The evaluation of the virucidal activity of HOCl was carried out after 10 min of exposition, on surfaces previously contaminated with the viruses Coronavirus 229E, ssRNA (+) virus, with the envelope and Adenovirus V, DNA virus not-enveloped [33].

**Figure 6:**
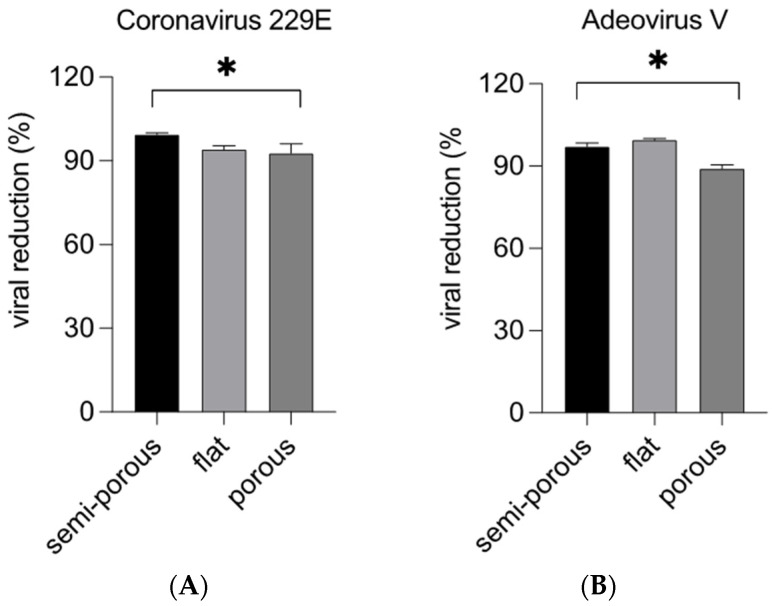
Virucidal effect of hypochlorous solution against Coronavirus 229E (**A** **panel**) and Adenovirus V (**B** **panel**) 1 × 10^7^ pfu/mL. HCoV-229E and Adenovirus V were incubated in the different surfaces and subjected to nebulization with a concentration of 300 ppm of HOCl. The nebulization time was set to 3 min and the exposure times 10 min. Data represent the percentage of viral reduction. Values were obtained by comparing the results with the virus control and represent the mean of 3 independent experiments. * *p* < 0.05.

The results obtained allow us to establish that HOCl at 300ppm used in aerosolized form has a virucidal capacity on envelope (Coronavirus 229E) and non-envelope (Adenovirus V) viruses; in fact, as we can see from Figure 6A,B, we observe good values of viral growth reduction after treatment with HOCl at 300 ppm. In fact, for Coronavirus 229E, we observe 99.9% of reduction in viral growth on a semi-porous surface, 95% for flat and 95% for a porous surface (Figure 6A and Table 3). Similar results were also obtained by evaluating the virucidal activity against Adenovirus V, we observed the ability of the hypochlorous solution 300 ppm to inactivate the virus. In this case the range of the reduction in viral growth was between 98% for semi-porous surface, and 99.9–90% for flat and porous surfaces, respectively (Figure 6B and Table 4). The values obtained from the virucidal test against Coronavirus 229E and Adenovirus V are highly significant.

### 3.5. HOCl Nebulization Did Not Affect the Viability of Epiderm and Epiairway Tissues

**Figure 7:**
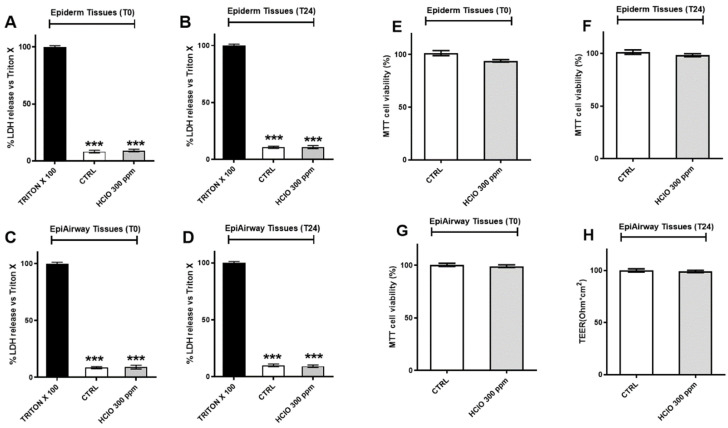
Effects of HOCl nebulization on Epiderm and Epiairway tissues. **Left panels** (**A**–**D**) Cytotoxicity evaluation by lactate dehydrogenase release in reconstructed human epidermis tissues (RHE) and Epiairway tissues maintenance media immediately after or after 24 h from the exposure to 300 ppm HOCl measured by an enzymatic assay. **Right panels** (**E**–**H**) cytotoxicity evaluation by MTT assay in reconstructed human epidermis tissues (RHE) and Epiairway tissues immediately after or after 24 h from the exposure to 300 ppm HOCl. Data are presented as mean  ±  SEM of four different experiments. *** *p* < 0.0001.

Once we demonstrated the bactericidal and viricidal effects on 300 ppm HOCl, we further analyzed the possible toxicity of our treatment on two 3D models: Epiderm and Epiairway tissues. As depicted in Figure 7A–D (left panels) the lactate dehydrogenase (LDH) secretion did not increase after the exposure to HOCl, at both T0 and T24 h, indicating that this dose did not affect the structure of plasma membrane. The safety of HOCl in both 3D tissue models was also confirmed by the MTT assay (Figure 7E–H, right panels) at both 0 and 24h time points.

### 3.6. HOCl Nebulization Does Not Alter Barrier Integrity and Morphology of Cutaneous and Respiratory 3D Tissues

**Figure 8:**
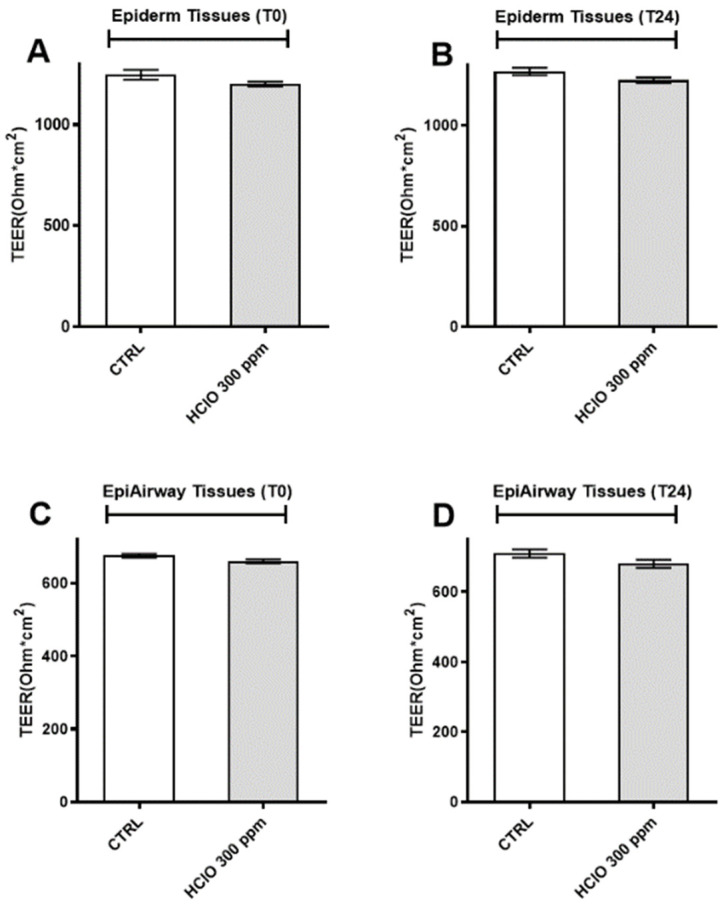
Effects of HOCl nebulization on Epiderm and Epiairway barrier function. (**A**,**B**) TEER value of Epiderm tissue immediately after, and after 24 h of 300 ppm HOCl nebulization. (**C**,**D**) TEER value of Epiairway tissue immediately after, and after 24 h of 300 ppm HOCl nebulization. Data are expressed as mean ± SEM of four different experiments.

**Figure 9:**
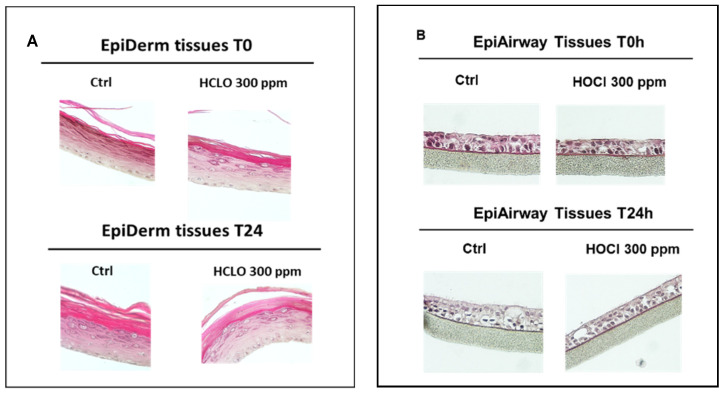
Epiderm (**A**) and Epiairway (**B**) tissue morphology evaluation by hematoxylin–eosin staining in control tissues and in tissues immediately after or 24 h after 300 ppm HOCL nebulization. Magnification 40×.

To investigate the effect of HOCl nebulization on barrier integrity of the selected tissues, the values of TEER were measured at time 0 and 24 h after the nebulization. As depicted in Figure 8 for both Epiderm (A,B) and Epiairway (C,D) tissues, the TEER value did not change after the exposure to HOCl, indicating that the selected dose did not affect the barrier integrity of the tissues. Then, to evaluate the possible effects of HOCl nebulization on tissues morphology, Ematossilin and Eosin staining was performed. As depicted in Figure 9 we did not observe any morphological changes in both tissue models, Epiderm (Figure 9A) and Epiairway tissues (Figure 9B), at any of the time points analyzed (T0 and T24). Concerning Epiderm tissue (Figure 9A), after 300 ppm HOCl nebulization, we observed an intact structure of the epidermis with basal, spinous, granulous and cornified layers well represented over the experimental procedure. In detail, the stratum basal, containing cuboidal mitotically active cells, was comparable in treated or non-treated tissues. Concerning the stratum spinosum, its grainy appearance, and the typical number of cell layers (three to five) were similar in nebulized and non-nebulized samples Finally, the outermost layer of the epidermis, the stratum corneum, did not display evident differences in nebulized or non-nebulized tissues.

Even in the case of Epiairway tissues (Figure 9B), after the exposure to 300 ppm HOCl, the 3D models maintained the typical structure of the respiratory tissue with ciliated apical surface and mucociliary epithelium without significant differences with respect to the control tissue.

**Figure 10:**
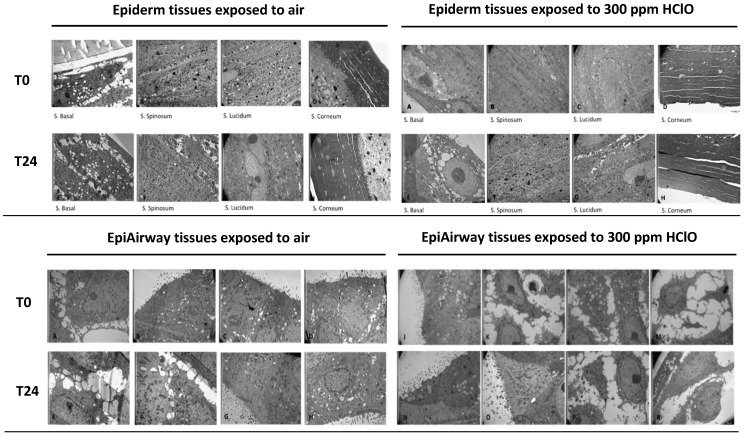
**Upper panels**: Epiderm morphology analyzed by TEM microscopy. (**Left panels**: **A**–**D**): TEM images of Epiderm tissues exposed to air (T0h); basal layer with large intercellular spaces, compact inner layer cells; stratum corneum without discontinuity; (**E**–**H**): TEM images of Epiderm tissues exposed to air (T24h); basal layer with large intercellular spaces, cells often vacuolized, loss of adhesion with the support; cells of the inner layers appear compact and stratum corneum compacted in many places, 5K magnification; (**Right panels**: **A**–**D**): TEM images of Epiderm tissues exposed to 300 ppm HOCl (T0h); compact basal layer and also inner layers; thick horny layer; (**E**–**H**): TEM images of Epiderm tissues exposed to 300 ppm HOCl (T24h); basal layer with large intercellular spaces, some vacuolized cells, detached from the support cells of the compact inner layers; stratum corneum with little areas of detachment, 5K magnification; **Below panels**: Epiairway morphology analyzed by TEM microscopy. (**Left panels**: **A**–**D**): TEM images of Epiairway tissues exposed to air (T0h); cells appear functionally normal, nuclei, mitochondria and surface normal; there is large intercellular spacing and some cells with numerous electron-dense lysosomal granules; (**E**–**H**): TEM images of Epiairway tissues exposed to air (T24h); basal cells with large intercellular spaces, detached from the support many in various necrotic steps, mitochondria dense with deposited material, superficial cells with less damaged but often vacuolized organelles rich in secretory granules, 5K magnification. (**Right panels**: **J**–**M**): TEM images of Epiairway tissues exposed to 300 ppm HOCl (T0h); the basal cells show large intercellular spaces, partially detached from the support, mitochondria dense with deposited material, superficial cells with normal ultrastructure (**N**–**R**): TEM images of Epiairway tissues exposed to 300 ppm HOCl (T24h); basal cells with large intercellular spaces, partially detached from the support, mitochondria dense with deposited material, superficial cells with less damaged but often vacuolized organelles rich in secretory granules, 5K magnification.

Finally, to better clarify the possible effects of HOCl nebulization on human tissues, the ultrastructural analysis by TEM was conducted in both Epiderm and Epiairway tissues after exposure to 300 ppm HOCl nebulization. The morphological aspect of the Epiderm tissue exposed to air does not show structural alterations as expected in either the deeper layers nor in the superficial stratum corneum which shows a uniform and compact structure up to 24 h upon the exposure (Figure 10).

The images obtained by TEM from exposing Epiderm tissues to 300 ppm HOCl (Figure 10A–H, right panels), show vacuolation of the basal layer and some detachment in the lamellae of the stratum corneum, but without any significant differences with the tissues exposed to air (Figure 10A–H, left panels) even after 24 h from the exposure.

The effects of the treatment after T0h and T24h of the Epiairway exposed to HOCl shown in Figure 10J–R (right panels), demonstrate that the superficial cells maintain a normal morphological appearance, if compared to tissues exposed to air (left panels); only in the deep layer, detachment between the cells is observed with the formation of intercellular spaces without nuclear and mitochondrial alterations.

## 4. Discussion

HOCl is classified within a group of molecules known as reactive oxygen species with an extremely powerful oxidation potential, which can participate in subsequent non-enzymatic reactions, such as the oxidation and chlorination of cellular components. The usual chemical behavior of HOCl with the carbon and nitrogen of the amino group is as an electrophilic agent, in which the chlorine atom combines with a pair of electrons from the substrate, while the hydroxide ion is simultaneously or subsequently separated, often assisted by a proton from the solvent or from a reactive center elsewhere in the substrate. This feature mainly occurs in reactions with ammonia nitrogen, amides, amino acids, and peptides, with phenolic and other aromatic compounds. Chloride ion transfer is favored by the negative charge, basicity, and nucleophilicity of the receiving agent. HOCl is known to rapidly react with biomolecules, and no specific enzymatic mechanism is known to remove these oxidants. Phospholipids, fatty acids, sterols, and sphingolipids are also susceptible to HOCl oxidation. Another lipid target of HOCl is the primary amino groups present in ethanolamine and serine glycerophospholipids [34].

The aim of our work was to provide information regarding disinfection of indoor environments using EW solution, a relatively well-studied compound, largely used as liquid biocide in many industries, from the food sector to healthcare applications, including chronic wound care disinfection. Although less studied, the use of HOCl as dry fog has shown potential benefit to disinfect indoor spaces where aerosols can be airborne for extended periods. Additionally, the COVID-19 virus can persist on inanimate surfaces for up to 9 days, and the periodical disinfection of indoor spaces could reflect the benefits on all surfaces in the room, contributing to reduce transmission [35]. Aside from an extensive selection of disinfectants against coronaviruses being certified by the EPA, there has not been an ideal disinfectant until now [36].

As recently reported, the World Health Organization (WHO) has requested to add HOCl to the core Essential Medicines List as a disinfectant, antiseptic and wound care agent because it has emerged in the current pandemic as the most potent and environmentally safe disinfectant available and with a wide range of efficacy against many human pathogens, in the range 180–460 ppm [37].

Starting from this evidence, in the present work, we have first established the optimal dose of HOCl to be used in the experimental protocols; the experimental results obtained on a well-studied 2D cellular model demonstrated that 24 h of exposure to 300 ppm of aerosolized HOCl did not affect cell viability, suggesting that 30-min nebulization is not toxic to cells. To expand our analysis, we evaluated the effects of aerosolization on cell morphology by scanning electron microscope (SEM) and transmission (TEM) analysis. Based on the experimental evidence also in this case, we can conclude that HOCl at 300 ppm does not cause damage to cell morphology, neither immediately after nebulization, nor after 24 h.

In consideration of the potential capacity of microorganisms to survive on surfaces, it is good practice to proceed frequently and to sanitize (cleaning and/or disinfect) the surfaces, operations which must be more accurate and regular for surfaces with high contact frequency. In the choice of multipurpose detergents and disinfectants it is necessary to consider a series of requirements, such as rapid action and long persistence of activity, biocidal activity, broadest possible spectrum of action, less danger at the concentrations of use for humans and on the materials to be treated, ease of application, quality and safety, cost-effectiveness of management, and non-induction to resistances. The WHO recommends “to ensure that environmental cleaning and disinfection procedures are followed consistently and correctly” [38]. Contamination of frequent touch surfaces in healthcare settings are therefore a potential source of viral transmission.

The air and surfaces of equipment, work surfaces, and work clothes, as well as hands, can represent important vehicles of microbiological contamination and potential sources of transmission of infectious agents. Numerous studies have highlighted the role of the inanimate environment in the epidemiology of infections caused by pathogenic bacteria, such as methicillin-resistant *Staphylococcus aureus* (MRSA), *Enterococcus* spp. vancomycin resistant (VRE), etc. Studies carried out on the persistence of microbes on surfaces have shown that these microorganisms are able to survive on dry surfaces for hours or days and in some cases for months [39]. Infections caused by Gram-negative and Gram-positive bacteria are frequently occurring. *Escherichia coli* is a well-studied model organism for Gram-negative bacteria. Survival analyses of *E. coli* on several inanimate surface have been published in numerous amounts. Different authors show *E. coli* survival on different inanimate surfaces. It is assumed that *E. coli* persistence is dependent on several factors. Next to strain specific characteristics and material nature, environmental conditions, such as temperature and humidity, have been shown to exhibit a huge impact on survivability duration. In contrast to *S. aureus*, where all bacterial strains survived on ceramic floors, synthetic fiber, cotton, and mattresses for more than eight weeks, *E. coli* was not so stable on all materials. *Pseudomonas aeruginosa*, Gram-negative bacteria, is associated with hospital acquired infections due to its capability to survive under challenging environmental conditions; it can survive on different inanimate surfaces in a material, species, and strain dependent manner.

As with bacteria, viral species also exhibit a different resistance on inanimate surfaces. The danger of viruses has been confirmed both by epidemiological evidence and by microbiological evidence that detect the presence of them on a large variety of surfaces. Viruses are able to resist various environmental factors and disinfection techniques, even those that are prolonged over time. The most important characteristic to keep in mind when evaluating the environmental resistance of viruses is of the “biological” type. The absence of an envelope gives them greater resistance to drying, making them more stable in the environment. It possesses a high genetic variability and a high recombination capacity that induce the development of dangerous viral agents that can stress unexpected health emergencies, such as that of the SARS-CoV-2 virus. *Coronaviridae* consist of positive single strand RNA viruses with an envelope, contributing to its name (from lat. corona—crown). There are seven human pathogenic strains of coronaviruses (CoV)—four endemic strains (229E, OC43, NL63, and HKU1) and three epidemic strains (severe acute respiratory syndrome coronavirus (SARS-1-CoV), Middle East respiratory syndrome coronavirus (MERS-CoV), severe acute respiratory syndrome coronavirus type 2 (SARS-CoV-2)). The mode of transmission is mainly by respiratory droplets and aerosols, but a certain contribution of contact transmission, e.g., via fomites, seems likely [40]. The family *Adenoviridae* comprises nonenveloped viruses with an icosahedral nucleocapsid containing a double stranded DNA genome, causing a wide range of illnesses, from mild respiratory tract infections or conjunctivitis to life-threatening multi-organ disease in patients with a weakened immune system. In addition, the survival of viruses on surfaces depends on many factors, the type of surface and its composition, the environment, the conditions of analysis as well as the period [41].

The efficiency of the transfer of a pathogen from surfaces to the skin is an important parameter for understanding transmission capacity and implementing effective hygiene measures. In the current study, we were able to demonstrate that aerosolized 300 ppm of HOCl had powerful and rapid killing properties on common bacteria (*E. coli*, *S. aureus* and *P. aeruginosa*) and viruses (Coronavirus 229E and Adenovirus V) on various surfaces (semi-porous, flat and porous).

Our study focuses on the use of HOCl solution in nebulized form for disinfection against microorganisms and surfaces of different nature to best estimate the bactericidal and virucidal activity of the HOCl. Nebulization time of 3 min and exposure time of 10 min showed excellent bacterial and viral growth reduction values on all microorganisms used.

It must be taken into account that these tests have been carried out starting from a stock of HCoV-229E and Adenovirus V with a high titer, and that, on average, the viral load found in aerosols or on surfaces may be significantly similar.

The results obtained show that the diversity of the materials after treatment does not affect the ability of the product to neutralize microorganisms. The values obtained as percentages of bacterial or viral growth reduction are in the range of 90–99.9%, values considered significant.

While most disinfectants are tested or studied in their liquid form on several types of surfaces, the only studies of viricidal/bactericidal efficacy of nebulized HOCl have been carried out when used as wet spray. Hakim et al. [42] present a comprehensive insight into the efficacy of HOCl in spray form to inactivate *Escherichia coli* and *Salmonella* with an effective concentration of 50–100 ppm on rayon fabric. Naka et al. [43] found that HOCl exhibited higher bactericidal activity compared with NaOCl with the same concentration of 0.5 ppm. Okamoto et al. [44] also showed that 20-min exposure to sprayed HOCl of 0.01% was sufficient to reduce 99.5% of *Staphylococcus epidermidis.*

Block and Rowan [15] also found the effective concentration of HOCl to be 200 ppm in decontaminating inert surfaces carrying noroviruses and other enteric viruses in a 1-min contact time. When diluted 10-fold, HOCl solutions at 20 ppm were still effective in decontaminating environmental surfaces carrying viruses in a 10-min contact time.

Air disinfection and hand hygiene are the major preventive measure in containing infections. High-risk exposed surface areas need to be cleaned frequently with a suitable disinfectant product [45]. The characteristic features of an ideal disinfectant are a low contact time with significant bactericidal and viricidal activity with it being safe for humans, the environment, surfaces, and equipment.

The amount of active chlorine declined markedly in aerosolized HOCl droplets, due to air and light exposure, falling by up to 60% by the time they cover the distance between the nozzle and the space around [46]. Despite fairly low concentration of HOCl used in the present work, we were able to clearly demonstrate antimicrobial and antivirus activities, making our discoveries particularly relevant to human daily life [47]. Solutions of HOCl have been shown to rapidly inactivate pathogens and viruses in test systems comparable to those used in this study [48]. Based on those results, commercial products containing HOCl have been recently placed on commerce and approved by the US Environmental Protection Agency (EPA), EU Chemical Agency (ECHA) and Australian Therapeutic Goods Administration (TGA), as a disinfectant against COVID-19.

More broadly, we note that widespread, high-volume use of disinfectants has come about as a response to the current pandemic, and it is becoming increasingly apparent that many of these, namely ozone or hydrogen peroxide, are inappropriate for such use patterns, never having been designed or intended for intensive applications around—and sometimes upon—humans. Toxicological evidence of serious untoward effects of repeated exposures to commonplace disinfectants, especially by inhalation of aerosols, are emerging [10]. Formaldehyde- and glutaraldehyde-based disinfectants are noxious and cytotoxic and may pose a serious health risk in addition to adversely affecting the environment [49]. Sodium hypochlorite-based disinfectants have severe adverse effects and cause skin irritation, membrane irritation, and acute toxicity [50]. Quaternary ammonium-based disinfectants irritate the skin and mucous membranes and are related to the development of airway allergies as well as induce occupational asthma and contact dermatitis [51]. For alcohol-based disinfectants and hand sanitizers, frequent use of high-concentration formulations may lead to skin damage or contact dermatitis with skin irritation, dryness, redness, and cracking [52]. Additionally, all of them may negatively impact the environment, soil, aquatic organisms, and wildlife, leading to serious and dramatic future consequences. In our study, the usage of 3D models helped us to further validate the 2D cell results concerning the lack of harmful effects of HOCl nebulization for human health. Indeed, 3D cell culture represents a more physiological approach closer to the morphology and physiology of the human tissues and therefore better mimicking the in vivo conditions. *Epiderm^®^* and *Epiairway*^®^, resembling cutaneous and respiratory tract tissues, respectively, were not affected by 300 ppm of HOCl exposure at the morphologically, metabolic rate and viability analyses. In addition, transepithelial electrical resistance (TEER) measurement confirmed that the nebulization did not induce any alteration of the cellular junctions, in either of the tissues.

Moreover, HOCl does not lead to the development of antimicrobial resistance, unlike genotoxic chemicals [53].

For its nature, HOCl is unstable because it rapidly decays to chloride, unless it is stored in dark and not exposed to air, preferably at 4 °C [54], ensuring a relatively short shelf-life, but it can be easily and inexpensively produced in situ combining an electrolysis and aerosolizing machine. A liter of HOCl can be manufactured only from sodium chloride, water and electricity, with an average cost of about EUR 0.25, considering the 2020 average European electric energy cost of 21.92 €cent/kWh [1]. Moreover, it avoids transportation of harmful products, such as chlorine gas and concentrated bulk hypochlorite, and it has been shown to hold significant economic advantage. In situ generation is of particular interest for developing countries, where the supply chain of bulk chemical commodities can be unreliable and accessing many locations is often a hurdle. Having cost-effective devices that could work in a standalone, solar-driven fashion, and generate chlorinated products on-site at low cost could help to improve the life of those communities [55,56].

## 5. Conclusions

The COVID-19 pandemic has caused both massive healthcare and economic disruption across the world. The current unavailability of an effective antiviral drug and the strong dependence on people mass vaccination mean that the implementation of effective preventive measures is necessary to counteract the coronavirus spread. The findings of this study have brought HOCl to the fore as a safe, yet highly effective, alternative to quaternary ammonium formulations, hypochlorite solutions, ozone, peroxide, and alcohols of various types.

Collectively these results demonstrated that our treatment can be considered as a natural and green disinfectant effective in killing bacteria and viruses and appears to be safe for human health. Moreover, we were able to provide reliable results without the need to use animal models in accordance with the Environmental Protection Agency (EPA) that supports measures dedicated to reducing them in toxicity testing.

## Figures and Tables

**Table 1:** The table shows the percentage of inhibition growth of individual microorganisms on different surfaces, after nebulization with HOCl at 300 ppm. All the data reported are the average of three independent experiments conducted (each experiment) in triplicate.

	*E. coli* (R%)	*S. aureus* (R%)	*P. aeruginosa* (R%)
Semi-porous nebulized	69	99.46	99.3
Flat nebulized	99.5	93	99.9
Porous nebulized	98.5	99.43	96

**Table 2:** The table shows the percentage of inhibition growth of mix of microorganisms on different surfaces, after nebulization with HOCl at 300 ppm. All the data reported are the average of three independent experiments conducted (each experiment) in triplicate.

	Mix Bacteria (R%)
Semi-porous nebulized	99.9
Flat nebulized	99.9
Porous nebulized	99.3

**Table 3:** Percentage values of plaque reduction after nebulization with hypochlorous solution at 300 ppm on different surfaces contaminated with Coronavirus 229E. All the data reported are the average of three independent experiments conducted (each experiment) in triplicate.

	Coronavirus 229E (R%)
Semi-porous nebulized	99.9
Flat nebulized	95
Porous nebulized	95

**Table 4:** Percentage values of plaque reduction after nebulization with hypochlorous solution at 300 ppm on different surfaces contaminated with Adenovirus V. All the data reported are the average of three independent experiments conducted (each experiment) in triplicate.

	Adenovirus V (R%)
Semi-porous nebulized	98
Flat nebulized	99.9
Porous nebulized	90

## Data Availability

Not applicable.
